# Natural Antioxidant Control of Neuropathic Pain—Exploring the Role of Mitochondrial SIRT3 Pathway

**DOI:** 10.3390/antiox9111103

**Published:** 2020-11-09

**Authors:** Sara Ilari, Luigino Antonio Giancotti, Filomena Lauro, Micaela Gliozzi, Valentina Malafoglia, Ernesto Palma, Marco Tafani, Matteo Antonio Russo, Carlo Tomino, Massimo Fini, Daniela Salvemini, Vincenzo Mollace, Carolina Muscoli

**Affiliations:** 1 Department of Health Sciences, Institute of Research for Food Safety & Health (IRC-FSH), University “Magna Graecia” of Catanzaro, 88201 Catanzaro, Italy; sara.ilari@hotmail.it (S.I.); micaela.gliozzi@gmail.com (M.G.); palma@unicz.it (E.P.); mollace@libero.it (V.M.); 2 Department of Pharmacology and Physiology, Henry and Amelia Nasrallah Center for Neuroscience, Saint Louis University School of Medicine, St. Louis, MO 63104, USA; luigi.giancotti@health.slu.edu (L.A.G.); milena.lauro@health.slu.edu (F.L.); daniela.salvemini@health.slu.edu (D.S.); 3 Institute for Research on Pain, ISAL Foundation, 47922 RN Torre Pedrera, Italy; valentinamalafoglia@yahoo.it; 4 Department of Experimental Medicine, University of Rome “Sapienza”, 00161 Rome, Italy; marco.tafani@uniroma1.it; 5 MEBIC Consortium, San Raffaele Rome Open University, 00163 Rome, Italy; matteo.russo@sanraffaele.it; 6 Scientific Direction, IRCSS San Raffaele Pisana, 00163 Rome, Italy; carlo.tomino@sanraffaele.it (C.T.); massimo.fini@sanraffaele.it (M.F.)

**Keywords:** neuropathic pain, natural antioxidants, polyphenols, sirtuin 3, mitochondria, oxidative stress, spinal cord

## Abstract

Neuropathic pain is a chronic painful disease. Data have shown that reactive oxygen species (ROS) are implicated in chronic pain. Particularly, the enhanced ROS production alters the mitochondrial genome and proteome through the accumulation of lipid peroxidation products, such as 4-hydroxynonenal (4-HNE) and malondialdehyde (MDA). Sirtuin 3 (SIRT3) is a mitochondrial protein and its activity can reduce ROS levels by modulating key antioxidant enzymes, such as manganese superoxide dismutase (MnSOD). Here, we evaluated the role of SIRT3 in the maintenance of basal levels of ROS in a model of chronic constriction injury (CCI) of the sciatic nerve and the protective effects of a natural antioxidant, the bergamot polyphenolic fraction (BPF). Rats were exposed to CCI of the sciatic nerve in the presence or absence of BPF (25–75 mg/kg). Level of acetylation, post-translational modulation on cysteine residues of proteins by HNE and SIRT3 activation, were detected in the spinal cord through western blotting, WES methodology and enzymatic assays. Our results reported that SIRT3 carbonylation and therefore its inactivation contributes to mitochondrial MnSOD hyperacetylation during CCI induced neuropathic pain in rats. In particular, we have demonstrated a close relation between oxidative stress, hyperalgesia, allodynia and sirtuins inactivation reverted by BPF administration.

## 1. Introduction

Neuropathic pain is the most common clinical manifestation of peripheral neuropathy, resulting from diabetes, infections, nerve-related diseases and drugs; the prevalence of neuropathic pain is almost 19% of adult Europeans [1,2,3,4].

Peripheral nerve injuries are often the cause of the onset of persistent pain indicating a disease progression process, which can result in chronic pain [2]. Knowledge on the processes underlying neuropathic pain has evolved in recent years indicating a complex scenario in which multiple cellular and molecular players contribute to the development of the disease [2]. Increasing evidence suggests that the action of oxidative species may be critical to the pain of several etiologies including neuropathic pain [5,6,7,8].

It is well known that neuropathic pain produces thermal hyperalgesia and allodynia contributing to the development of reactive oxygen and nitrogen species (ROS; RNS) and cytokines, able to carry out oxidative, nitrosative and nitrative activities implicated in persistent pain states [9,10,11,12,13]. Abnormalities of inflammatory cytokine profile play an important role in neuropathic pain. A recent study [14] has shown that the abnormal activation of pro-inflammatory cytokines, such as TNFα and ILß, produced in both spinal cord and in medial prefrontal cortex of a rat model of Spared Nerve Injury, were responsible of Chronic Neuropathic Pain - related anhedonia [14].

In addition, over-production of nitroxidative species results in oxidative stress and may lead to irreversible functional damages, including cancer, diabetes, obesity, osteoarthritis [15,16,17].

Oxidative stress causes an alteration in mitochondrial dynamics leading, in turn, to the increase of ROS production, responsible for the dysfunctional mitochondria [18,19,20,21,22,23]. Numerous studies have demonstrated new mitochondrial regulation pathways in cellular oxidative stress and inflammatory signaling [9,24,25,26]. In particular, it has been shown that oxidative damage, through the accumulation of lipid peroxidation (4-hydroxynonenal (4-HNE), malondialdehyde (MDA), is responsible for post-translational modification and inactivation of key mitochondrial proteins, by altering mitochondrial activities in cardiovascular, neurodegenerative diseases and pain [21,27,28,29,30,31,32].

Recent data have indicated that mitochondrial dysfunction is one of the causes of neuropathic pain contributing to the disease progression [33,34]. Considering that approximately 20% of mitochondrial proteins are acetylated, acetylation/deacetylation appears as a pivotal regulator mechanism for mitochondrial proteome function [35].

Interestingly, Sirtuin 3 (SIRT3) can improve the ability of mitochondria to reduce ROS levels and protect against oxidative stress by regulating the activity of key antioxidant enzymes, such as manganese superoxide dismutase (MnSOD) [19,36,37,38,39]; indeed, SIRT3 is a mitochondrial enzyme that regulates the mitochondrial lysine acetylation in an NAD+-dependent manner [38].

Moreover, SIRT3 plays an important role in a wide range of biological activities such as energy homeostasis, apoptosis, inflammation and it can be used for protection against cardiovascular diseases and heart failure [40,41,42]. Also, SIRT3 deacetylation targets are implicated in the oxidative stress response [40,41,42], regulating acetylation and activity of key antioxidant enzymes to prevent oxidative stress [43,44]. Several studies have described pharmacological approaches leading to SIRT3 activation, for the treatment of diseases associated with SIRT3 deficiency [45] and several aging-related conditions, such as type 2 diabetes, most persistent diseases of rheumatologic interest or chronic lymphocytic leukemia [45,46]. The mechanism by which SIRT3 regulates the antioxidant-driven redox signaling is still unclear and represents an interesting area of research.

Our recent data revealed the involvement of mitochondrial SIRT3 during carrageenan-induced inflammatory pain [19]. We spotted as SIRT3 inactivation, induced by oxidative stress condition, could be responsible for MnSOD inactivation with continuous ROS production that leads, in turn, to mitochondrial dysfunction [19]. Removal of free radicals by natural or synthetic antioxidants blocks protein post-translational modification, attenuating the observed biochemical changes and preventing the development of pain [32,47,48,49].

In this regard, it has been observed that polyphenols, such as resveratrol, bergamot, hydroxytyrosol and oleuropein, have strong antioxidant and anti-inflammatory properties [19,21,32,50,51]. Moreover, these substances can activate sirtuins directly or indirectly in several animal models [50]. Therefore, modulation of glutamatergic transmission and sirtuins’ activation by polyphenols could be considered as new strategies in chronic pain therapy.

Studies showed that bergamot (in particular the bergamot polyphenolic fraction; BPF) has a higher concentration of polyphenols compared to other citrus fruits; in fact, it contains 5 main flavonoids namely neoeriocitrin, naringin, neohesperidin, melitidin and brutieridin [32,52,53,54].

Particularly, bergamot polyphenols have hypolipemic and hypoglycemic activity: the oral assumption, for 30 days, reduces total cholesterol, LDL (an effect also accompanied by elevation of HDL) and triglyceride levels in both rats and human patients [52].

The co-administration of morphine and BPF can be a good therapeutic approach for the treatment of chronic pain thanks to its ability to inhibit, in a dose-dependent manner, the development of tolerance preserving analgesic-morphine property [32].

Based on these data, here, we propose that, in the chronic constriction injury (CCI) model of neuropathic pain, SIRT3 modifications can alter mitochondrial homeostasis contributing to the insurgence and maintenance of pain.

## 2. Materials and Methods

### 2.1. Animals

8 weeks old males rats (Sprague-Dawley, 225−250 g, Envigo), were used according to the Italian regulations for animal protection in experimental and other scientific purposes (D.L. 26/2014), the European Economic Community regulations 2010/63/UE (authorization number 577-2016-PR) and the NIH Guideline on Laboratory Animal Welfare. The numbers of animals used are the minimum number necessary to achieve statistical significance at the *p* < 0.05 as set forth by the International Society for the Study of Pain guidelines. Rats (2 per cage) were housed and preserved fixed temperature (21 ± 1 °C) and humidity (60 ± 5%) conditions, allowed for food ad libitum, in a 12-hour light/12-hour dark cycle. Experiments were performed between 7:00 and 10:00 a.m. in a quiet room.

Natural antioxidant Bergamot polyphenolic fraction (BPF) was kindly provided by H&AD (Herbal and Antioxidants Derivatives srl). Unless specified, all drugs were purchased from Sigma Aldrich and dissolved in saline (sodium chloride 0.9%).

### 2.2. Induction of Neuropathic Pain

Chronic constrictive injury (CCI) to the sciatic nerve of the right hind paw in rats was performed under general anesthesia, using the Bennett model [15]. Nociceptive thresholds were assessed before and after performing surgery, on days 0, 3, 7, 10, 14 and 21. Rats were sacrificed 14 or 21 days after the CCI; the dorsal half of the spinal cord lumbar region enlargement (L4–L5) was excised and immediately frozen in liquid nitrogen for subsequent analysis.

### 2.3. Osmotic Pump Implantation

Male Sprague Dawley rats, were lightly anesthetized with Zoletil and Ronpum (1:1; i.p.) and were subcutaneously implanted (in the inter-scapular region) with primed osmotic minipumps (Alzet), to deliver drugs or saline at 2.5 µL/h over 21 days. The concentrations of BPF and pregabalin resulted in a daily dose of 25–75mg/kg and 10 mg/kg respectively. Mini pumps were filled according to manufacturer’s specifications. The use of the osmotic pump ensures a continuous subcutaneously delivery of drugs avoiding intermittent periods of withdrawal. The integrity of the pump delivery system was re-examined at the end of each experiment when the spinal cords are collected.

### 2.4. Experimental Groups

Animals were randomly assigned into the following groups:

- Sham group: Rats (*n* = 15) subjected to a surgical procedure to expose the sciatic nerve without any ligation and treated with saline concurrently with surgery (day 0), for 21 consecutive days.
- CCI group: Rats (*n* = 15) undergoing chronic constrictive injury (CCI) to the sciatic nerve and treated with saline (vehicle) concurrently with surgery (day 0), for 21 consecutive days.
- Drugs groups: Rats (*n* = 15 for each dose) subjected to CCI to the sciatic nerve and treated with different doses of BPF (25 mg/kg, 50 mg/kg or 75 mg/kg) or pregabalin (10 mg/kg) concurrently with surgery (day 0), for 21 consecutive days.

The dose of the compounds has been chosen according with the bibliography [32,51,55].

### 2.5. Behavioral Test

**Mechanical allodynia** of the hind paw was measured by determining withdrawal thresholds to a calibrated series of von Frey filaments. The paw withdrawal threshold (PWT) was determined by sequentially increasing and decreasing the stimulus strength (the “up- and-down” method). Each rat was placed in a Plexiglas cage with a metal mesh floor and allowed to acclimate for 15 min before testing. The filaments were applied perpendicularly to the medial plantar surface of the paw in an ascending order of bending force sufficient to bend the microfilament. Each filament was applied three times and each application lasted for two seconds. A 30-sec interval was set between each application. A positive response was defined as the paw withdrawal threshold (PWT), when rats showed at least three withdrawal responses out of five times application to a filament [15].

**Mechanical hyperalgesia** was assessed using the Randall–Selitto analgesiometer (Ugo-Basile, Varese, Italy). An incremental pressure was applied on the dorsal surface of paws and the pressure that exhibited the first nociceptive response such as squealing and reflex movement was recorded as the pain threshold for injured rats. The stimulus was applied on the paw with a cut off pressure of 250 g to avoid injury and damage. Results are expressed as Paw-withdrawal threshold (PWT (g)).

**Thermal hyperalgesia** was evaluated by radiant heat paw withdrawal test as described by Hargreaves [56] and a cut off latency of 20 sec was employed, in order to prevent tissue damage in non-responsive animals. Each rat was placed in a Plexiglas cage to acclimate for 15 min (Ugo-Basile, Varese, Italy). A mobile unit consisting of a high intensity projector bulb was positioned to deliver a thermal stimulus directly to an individual hind paw from beneath the chamber. The withdrawal latency period of paws was determined to the nearest 0.1 sec with an electronic clock circuit and thermocouple. If the animal failed to respond by 20 sec the test was terminated. Each point represents the delta change (sec) in withdrawal latency [withdrawal latency of left paw (contralateral paw) minus withdrawal latency of the right hind paw which received the chronic constrictive injury (CCI) to the sciatic nerve (ipsilateral paw)] at each time point. Results are expressed as Paw-withdrawal latency changes (sec).

### 2.6. Tissue Preparation for Mitochondrial Extraction

Lumbar spinal cord (L4-L6) tissues were homogenized with lysis buffer (250 mM Sucrose; 10 mM Tris; 1mM ethylenediaminetetraacetic acid (EDTA); 1% protease inhibitor cocktail; pH 7.8) at 1:3 *w/v* ratio. Extracts were centrifuged at 1000× *g* for 10 min. and the obtained supernatant were re-centrifuged at 12,000× *g* for 15 min. Lysis buffer (1% Triton; 1% protease inhibitor cocktail) was used to re-suspend the pellets. Protein concentration was detected using the Bicinchoninic Acid (BCA) protein assay (Pierce) after centrifugation at 10,000× *g* for 10 min at 4 °C. 

### 2.7. Immunoprecipitation and Western Blot Analysis

Mitochondrial fractions were used for immunoprecipitation and western blot analysis. 50 µL of protein A-sepharose 4B resin (Sigma, Milan, Italy, cat. P9424) was washed four times in PBS. Each wash was consisted by mixing fresh PBS with resin (beads) and then the collection of cleaned beads by centrifugation (14,000 rpm at 4 °C for 1 min). After lysis Buffer (1% Triton; 1:100 Protease inhibitor cocktail) re-suspension, cleaned resin was incubated with anti-SIRT3 antibody (1:1000; Cell Signaling, Milan, Italy) or anti-MnSOD (1:1000; Millipore, Milan, Italy), O/N at 4 °C, gently mixing. 250 µg of tissue homogenate supernatant were incubated with pre-washed beads (4 time in PBS) O/N at 4 °C. After centrifugation, pellets were used for Sodium Dodecyl Sulphate - PolyAcrylamide Gel Electrophoresis (SDS-PAGE) analysis.

Western blot analyses of immunoprecipitated proteins and total lysates were performed using specific antibodies. Briefly, samples were loaded in 12% SDS-PAGE minigels and then electrophoretically transferred to nitrocellulose membranes. 

Membranes were blocked (2 h, room temperature) with 1% Bovine Serum Albumin (BSA)/1× TBS/0.1% Tween-20. Membranes were incubated with rabbit polyclonal anti-Lys-Acetylated antibodies (O/N, 4 °C, 1:1000; Cell Signaling, Milan, Italy), mouse monoclonal anti-4HNE antibodies (O/N, 4 °C, 1:1000; R&D Systems, Milan, Italy), rabbit polyclonal anti-SIRT3 antibodies (O/N, 4 °C, 1:1000; Cell Signaling, Milan, Italt), rabbit polyclonal anti-MnSOD antibodies (O/N, 4 °C, 1:1000; Millipore, Milan, Italy) or mouse monoclonal anti-Prohibitin antibodies (O/N, 4 °C, 1:1000; Thermo Scientific, Milan, Italy). After washing with TBS/T, membranes were incubated with specific peroxidase-conjugated secondary antibody (1 h, room temperature, 1:10000 dilution; Amersham, GE Healthcare, UK) and the complex was detected by an enhanced chemiluminescence detection system (ECL, Amersham, UK). Protein levels quantitation was then performed by densitometry using Image Quant 5.2 software by Molecular Dynamics.

### 2.8. WES (Simple Western™)

Detection of 4-HNE adducts of histidine residues and total acetylation mitochondrial protein expression were determined using WES (Simple Western™) by Protein Simple©. Protein Simple© keeping the concept of western blot analysis, has changed the detection method. In fact, the Simple Western™ system uses capillary electrophoresis to identify and quantify a protein of interest. Simple Western assays are automated, capillary-based immunoassays. During this type of assay, protein samples are separated, immobilized and probed with target specific antibodies by automated steps. 

Initially, the capillary was filled with separation and stacking matrix. Then, reagents (Dithiothreitol, DTT; Fluorescent 5X Master Mix, Biotinylated Ladder) were prepared according to the protocol. Samples were diluted with 0.1X Sample Buffer (after dilution of 10X Sample Buffer 1:100 with water), combined with 5X Master Mix (1 MasterMix:4 Sample) to obtain 1.6 mg/mL and finally denatured. Antibodies were diluted to their optimal concentration (4-HNE 1:100 R&D System; Lys-Acetylated antibodies 1:100; Cell Signaling; TOM20 1:16000 Santa Cruz, Heidelberg, Germany) and Luminol (150 µL)-Peroxide (150 µL) mix was prepared. The plate was finally filled following the protocol scheme: (5 µL of Biotinylated Ladder, 5 µL of Samples, 10 µL of Wes Antibody Diluent II, 10 µL of Primary Antibody, 10 µL of Streptavidin-HRP, 10 µL of Secondary Antibody and 10 µL of Luminol-Peroxide Mix). Sample containing SDS, DTT and fluorescently labeled MW markers were taken from the plate and loaded into the capillaries. Voltage was applied across the capillaries to separate proteins in the sample. The capillaries were exposed to UV light, activating the proprietary linking chemistry and locking the separated proteins to the capillary wall. The capillaries were rinsed and immunoprobed for specific proteins with an HRP-labeled antibody. Luminol and peroxide were loaded into the capillaries to catalyze chemiluminescent light generation, which is captured by a charge-coupled device (CCD) camera. Simple Western™ systems captured data as a chemiluminescent image of the capillary. Images were analyzed by Compass software and could be presented as an electropherogram or as a lane.

### 2.9. Malonylaldehyde Detection

MDA quantification was performed through thiobarbituric acid reactive substances (TBARS) assay. In brief, tissue sample were added to a vial containing 10% NaOH, 20% Acetic Acid and TBA. The vials were boiled at 90–100 °C and after 1h the tubes were placed on ice to stop reaction. Before being transferred to a black 96-well microtiter plate samples were centrifugated 10 min at 1600× *g* at 4 °C. The MDA-TBA adduct was measured fluorometrically at an excitation wavelength of 530 nm and emission wavelength of 550 nm using Infinite 200 microplate fluorometer (Tecan, Männedorf Switzerland).

### 2.10. Determination of MnSOD Activity in the Spinal Cord

MnSOD activity was detected using Superoxide Dismutase Assay kit (Cayman Chemical) following the manufacturer’s protocol. The method uses tetrazolium salt to quantify superoxide radicals generated by xanthine oxidase and hypoxanthine. The standard curve was generated by using a quality-controlled SOD standard. The absorbance was monitored at 440–460 nm using a TECAN Sunrise Reader (Tecan, Männedorf, Switzerland). One unit of SOD is defined as the amount of enzyme needed to exhibit 50% dismutation of the superoxide radical. Enzymatic activity was presented as units/mL per milligram of total protein. The results were defined after triplicate analysis. 

### 2.11. Determination of Proteins Carbonylation in the Spinal Cord

Protein carbonyl groups were detected using the Oxyblot protein oxidation Kit (Millipore) following the manufacturer’s protocol. Briefly, carbonyl derivates of proteins were detected by reaction with 2,4-dinitrophenylhydrazine (DNPH) and the abundance of carbonyl protein groups was analyzed by Western Blot.

### 2.12. SIRT3 Deacetylase Activity Assay

SIRT3 deacetylase activity was determined using a SIRT3 Fluorimetric Activity Assay/Drug Discovery Kit (Biomol Research Laboratories) following the manufacturer’s protocol. In brief, the mitochondrial extract was incubated with the Fluor de Lys substrate buffer and then incubated with Fluor de Lys Developer. 

Infinite 200 microplate fluorometer (Tecan) were used to detect emitted light at 460 nm, after excitation at 360 nm.

### 2.13. Statistical Analysis

Data are presented as means ± SEM for n animals. Time course statistical analysis were performed via two-way repeated measures of variance (ANOVA) with Bonferroni comparisons.

After one-way ANOVA analysis, Tukey test was used for all remaining data. A threshold of 0.05 was considered for statistical significance. GraphPad Prism (v8.00; GraphPad Software, Inc, San Diego, CA, USA) was used for all the analysis.

## 3. Results

### 3.1. Natural Antioxidant Treatment Reduces Experimental Pain-Associated Behaviour in CCI Rats

**Figure 1:**
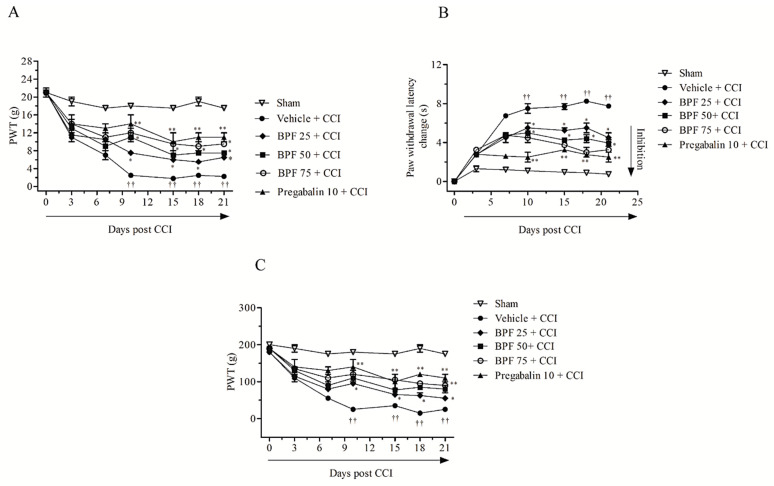
Effects of treatment with bergamot polyphenolic fraction (BPF) on allodynia and hyperalgesia after inducing Chronic Constriction Injury (CCI) in rats. In rats with CCI, subcutaneous infusion of BPF (25–75mg/kg) or pregabalin (10 mg/kg), concurrently with surgery (day 0), attenuated the development of mechano-allodynia (**A**), thermal hyperalgesia (**B**) and mechano-hyperalgesia (**C**) assessed over a 21 days period. Data are expressed as mean ± SEM for 15 rats in each group. * *p* < 0.05; ** *p* < 0.005 versus vehicle + CCI, ^††^ *p* < 0.05 versus sham.

The Chronic Constriction Injury (CCI) model of peripheral nerve provides a model system to investigate the effectiveness of potential therapeutic agents to modify chronic neuropathic pain. In rats, the CCI of the sciatic nerve leads to debilitating symptoms, such as spontaneous pain, hyperalgesia, allodynia and dysesthesia/paresthesia. Here, we observed that CCI causes a significant reduction in both mechanical withdrawal thresholds and thermal withdrawal latencies of the ipsilateral hind paw, indicating the presence of mechanical and thermal pain behavior as measured employing the Von Frey and Randall-Selitto test and the Hargreaves method respectively (Figure 1A–C). Injection of vehicle (saline) did not change the mechanical withdrawal threshold or thermal withdrawal latency (Figure 1A–C). No effects on contralateral paw withdrawal were found (Supplementary Figure S1).

Subcutaneous infusion of BPF (25–75 mg/kg) [32,51] by minipumps, concurrently with surgery (day 0) up to 21 days significantly modified, in a dose dependent manner, nociceptive parameters associated with time as follows: a significant enhancement in the mechanical withdrawal threshold and thermal withdrawal latencies were produced in the presence of BPF (25–75 mg/kg) starting from day 10 post injury up to day 21. Subcutaneous infusion of pregabalin (10 mg/kg [55], concurrently with surgery (day 0)) by minipumps, over the same timeframe, showed the same efficacy (Figure 1A–C). The drugs administration by minipump is essential ensure a continuous subcutaneously delivery of BPF or pregabalin avoiding intermittent periods of withdrawal.

### 3.2. CCI-Induced Hyperalgesia and Allodynia are Associated with an Increase of Lipid Peroxidation Products and Post-Translational Modification of Mitochondrial Proteins

**Figure 2:**
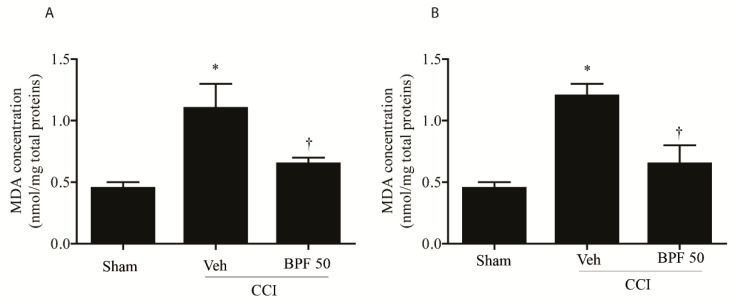
Malondialdehyde is associated with an increase of oxidative stress during neuropathic pain. Spinal malondialdehyde (MDA)indicates the presence of oxidative stress during chronic pain either 14 days (**A**) and 21 days (**B**) post injury. CCI rats showed a significant level of MDA either 14- and 21-days post injury. Subcutaneous infusion of BPF (50 mg/kg), concurrently with surgery (day 0), led to a significant reduction of these values. Results are expressed as mean ± SEM for 6 rats. * *p* < 0.05 compared to sham; ^†^ *p* < 0.05 compared to vehicle + CCI.

**Figure 3:**
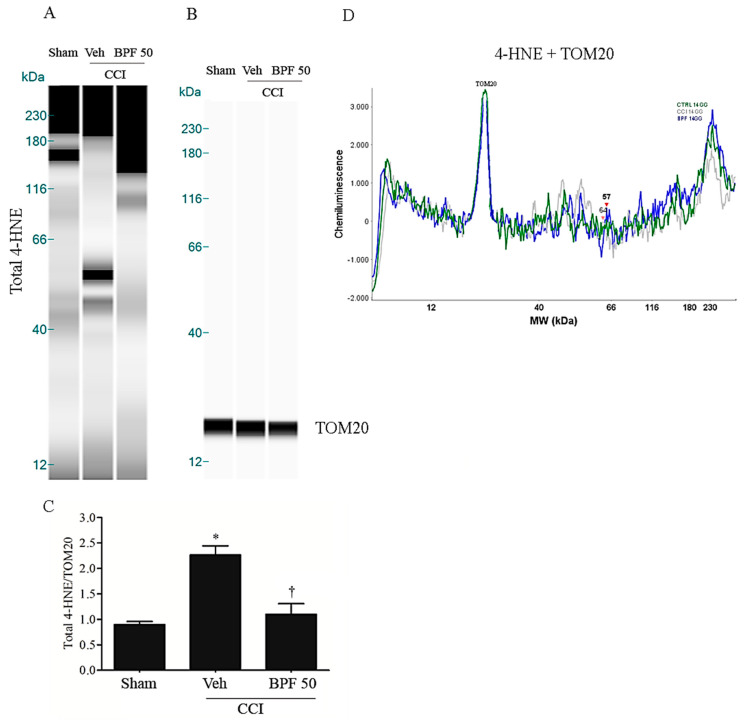
The development of hyperalgesia and allodynia coincided with an increase of 4-HNE level 14 days post injury. (**A**) CCI rats showed a significant production of 4-HNE in spinal cord 14 days post injury as measured by Simple Western (WES) methodology. Infusion of BPF (50 mg/kg), concurrently with surgery (day 0), showed a significant reduction of these values. (**B**) No difference for TOM20 expression was detected among the lanes in these conditions. (**A**,**B**) Lanes and (**D**) electropherogram are representative of results from 6 different animals. (**C**) Densitometric analyses of all animals per groups are reported. Results are expressed as mean ± SEM for 6 rats. * *p* < 0.05 versus sham; ^†^ *p* < 0.05 versus vehicle + CCI.

**Figure 4:**
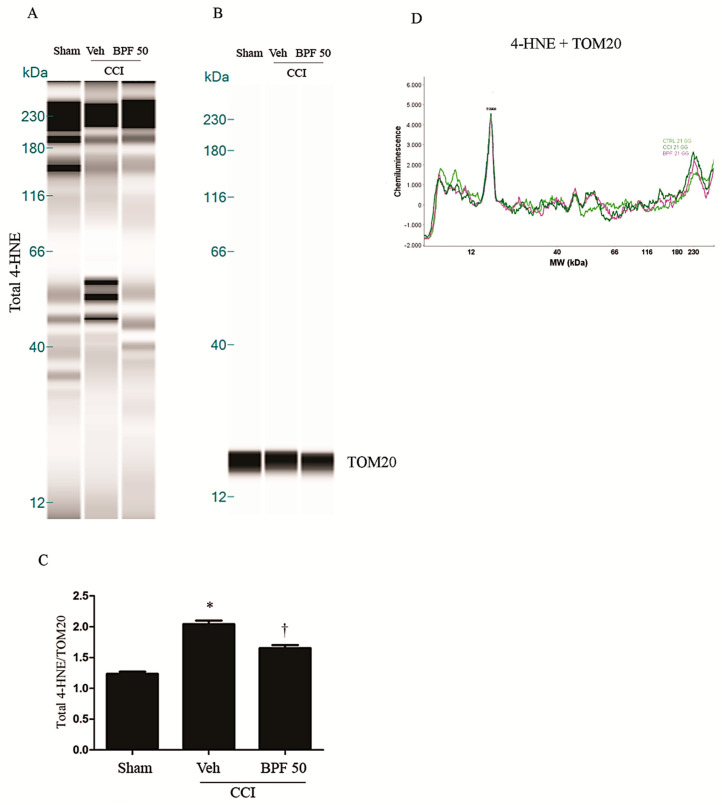
The development of hyperalgesia and allodynia coincided with an increase of 4-HNE level 21 days post injury. (**A**) CCI rats showed a significant production of 4-HNE in spinal cord 21 days post injury as measured by WES methodology. Subcutaneous infusion of BPF (50 mg/kg), concurrently with surgery (day 0), showed a significant reduction of these values. (**B**) No difference for TOM20 expression was detected among the lanes in these conditions. (**A**,**B**) Lanes and (**D**) electropherogram are representative of results from 6 different animals. (**C**) Densitometric analyses of all animals per groups are reported. Results are expressed as mean ± SEM for 6 rats. * *p* < 0.05 versus sham; ^†^ *p* < 0.05 versus vehicle + CCI.

CCI-induced neuropathic pain development corresponds to the increase of the level of spinal malondialdehyde (MDA) (Figure 2A,B) and 4-hydroxynonenal (4-HNE) (Figure 3 and Figure 4, Supplementary Figure S2) either 14- and 21-days post injury. 

These effects were attenuated, at the level of the spinal cord (L4-L5), by treatment with BPF at 50 mg/kg (intermediate dose with an antioxidant effect similar to the effect due to the highest dose we used) (Figure 2, Figure 3 and Figure 4 and Figure S2).

**Figure 5:**
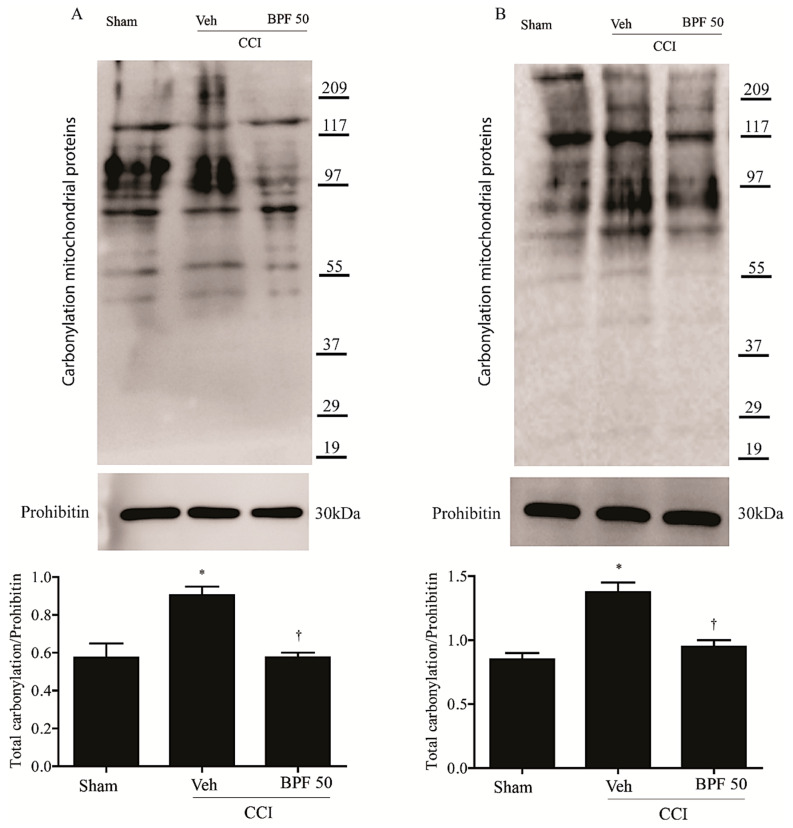
The increase of 4-HNE level cause carbonylation of mitochondrial proteins. High 4-HNE levels induced carbonylation of mitochondrial proteins either 14 days (**A**) and 21 days (**B**) post injury in CCI-only rats. Subcutaneous infusion of BPF (50 mg/kg), concurrently with surgery (day 0), prevented mitochondrial proteins carbonylation. In these conditions, prohibitin expression appeared statistically the same among groups. Gels are representative of results from 6 animals. Densitometric analyses of all animals per groups are reported. Results are expressed as mean ± SEM for 6 rats. * *p* < 0.05 compared to sham; ^†^ *p* < 0.05 compared to vehicle + CCI.

**Figure 6:**
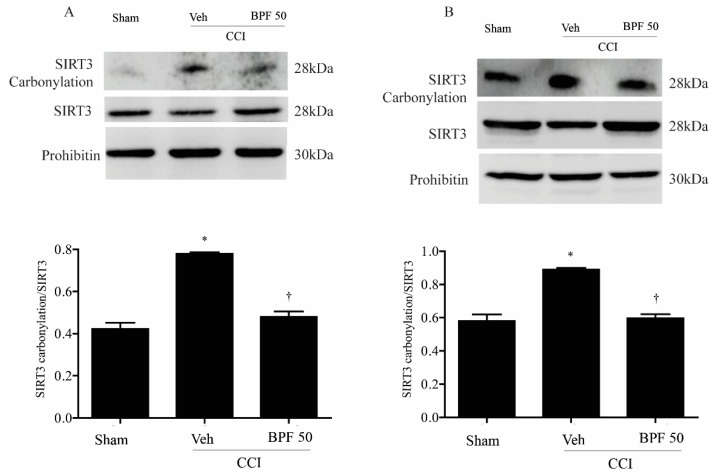
The development of hyperalgesia cause carbonylation of SIRT3 14 days post injury. Chronic constriction injury of the sciatic nerve (CCI) led to significant carbonylation of mitochondrial SIRT3 14 days (**A**) and 21 days (**B**) post injury, as measured by immunoprecipitation and Western blot. Infusion of BPF (50 mg/kg), concurrently with surgery (day 0), prevented SIRT3 carbonylation. In these conditions, prohibitin expression was statistically the same among the lanes. Gels are representative of results from 6 animals. Densitometric analyses of all animals per groups are reported. SIRT3 and SIRT3 carbonylation were first normalized with prohibitin and then these values were used to obtain carbonylation/total SIRT3 ratio. Results are expressed as mean ± SEM for 6 rats. * *p* < 0.05 compared to sham; ^†^ *p* < 0.05 compared to vehicle + CCI.

To measure the impairment of spinal mitochondrial protein due to oxidative stress through 4-HNE production, the carbonylation level of mitochondrial proteins and SIRT3 was analyzed by derivatizing immunoprecipitated SIRT3 with 2,4-dinitrophenyl hydrazine (DNPH). We observed an increase of mitochondrial carbonylated proteins (Figure 5A,B) and in particular SIRT3 carbonylation, either 14- and 21-days post CCI (Figure 6A,B).

The attenuation of mechanical and thermal hyperalgesia by BPF (50 mg/kg) is associated with a reduction of post-translational carbonylation of total mitochondrial proteins in the spinal cord (Figure 5A,B).

Furthermore, a relatively low level of carbonylation on SIRT3 in sham animals has been demonstrated (Figure 6). The CCI procedure induced an increase of SIRT3 carbonylation (Figure 6A,B) either 14 and 21 days post injury and the treatment with BPF (50 mg/kg) led to a significant reduction of SIRT3 carbonylation adducts (Figure 6A,B).

**Figure 7:**
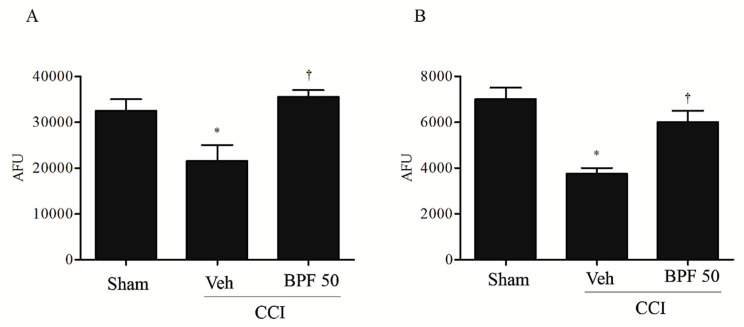
Carbonylation on SIRT3 is linked to inactivation of its biological function. Carbonylation and SIRT3 enzymatic activities intimately linked. In fact, animals with CCI, in comparison with sham group, presented low levels of SIRT3 activity either 14 days (**A**) and 21days (**B**) post injury as shown by western blotting results. Infusion of BPF (50 mg/kg), concurrently with surgery (day 0), restored SIRT3 activity. Results are expressed as mean ± SEM for 6 rats. * *p* < 0.05 compared to sham; ^†^ *p* < 0.05 compared to vehicle +CCI.

It is known that the carbonylation reaction alters the function of many proteins and may play a role in regulating SIRT3 activity [19]. We demonstrated that once carbonylated, mitochondrial SIRT3 is inactivated, losing its ability to deacetylate either 14 or 21 days after injury (Figure 7A,B); treatment of rats with BPF inhibited SIRT3 carbonylation, preventing its inactivation (Figure 7A,B).

### 3.3. Interaction between SIRT3 and Mitochondrial MnSOD Target Proteins

**Figure 8:**
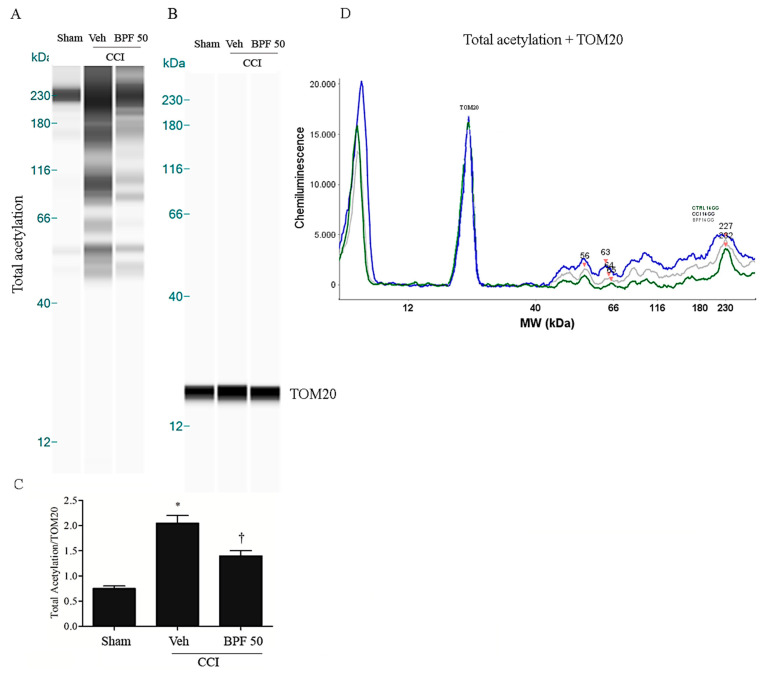
The inhibition of SIRT3 induces acetylation on mitochondrial proteins 14 days post injury. (**A**) Inactivation of SIRT3 led to significant acetylation of mitochondrial proteins 14 days post injury as shown by WES methodology. Subcutaneous infusion of BPF (50 mg/kg), concurrently with surgery (day 0), prevented mitochondrial proteins acetylation. (**B**) No difference for TOM20 expression was detected among the lanes in these conditions. (**A**,**B**) Lanes and (**D**) electropherogram are representative of results from 6 animals. (**C**) Densitometric analyses of all animals per groups are reported. Results are expressed as mean ± SEM for 6 rats. * *p* < 0.05 versus sham; ^†^ *p* < 0.05 versus vehicle + CCI.

**Figure 9:**
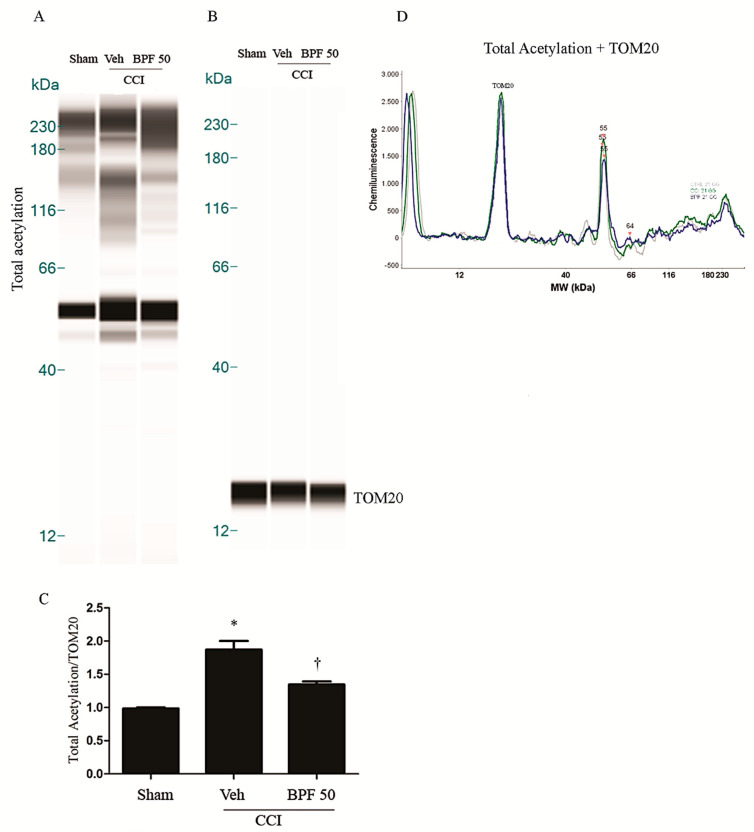
The inhibition of SIRT3 induces acetylation on mitochondrial proteins 21 days post injury. (**A**) Inactivation of SIRT3 led to significant acetylation of mitochondrial proteins either 21 days post injury as shown by WES methodology. Subcutaneous infusion of BPF (50 mg/kg), concurrently with surgery (day 0), prevented mitochondrial proteins acetylation. (**B**) No difference for TOM20 expression was detected among the lanes in these conditions. (**A**,**B**) Lanes and (**D**) electropherogram are representative of results from 6 animals. (**C**) Densitometric analyses of all animals per groups are reported. Results are expressed as mean ± SEM for 6 rats. * *p* < 0.05 versus sham; ^†^ *p* < 0.05 versus vehicle + CCI.

SIRT3 is considered a crucial protein for oxidative stress regulation, controlling the expression and function of many other mitochondrial proteins via deacetylation [43,57]. In the Figure 5, Figure 6 and Figure 7, we showed that carbonylation plays a role in regulating SIRT3 activity. Its inhibition may alter the function of several mitochondrial proteins: in the Figure 8; Figure 9, we observed a dramatic increase of mitochondrial protein acetylation in the spinal cord (L4-L5) of animals receiving CCI to the sciatic nerve, while rats treated with BPF (50 mg/kg) reduced the acetylation of mitochondrial proteins (Figure 8, Figure 9 and Supplementary Figure S3).

**Figure 10:**
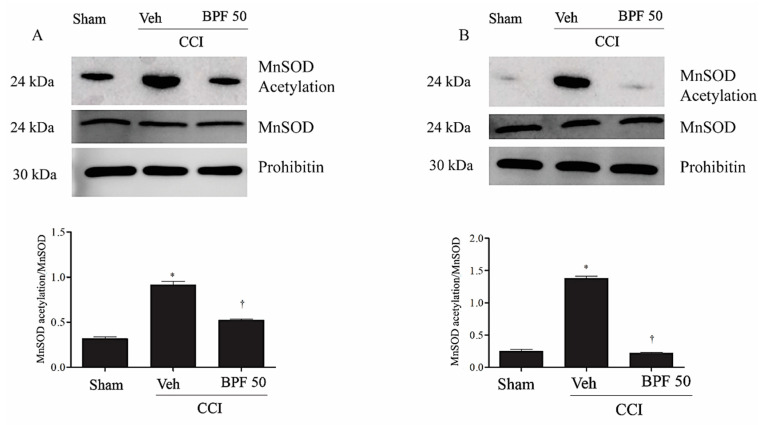
The inhibition of SIRT3 led to MnSOD acetylation. Inactivation of mitochondrial SIRT3 by the CCI injury induced mitochondrial MnSOD acetylation either 14 days (**A**) and 21 days (**B**) post injury as measured by immunoprecipitation. Subcutaneous infusion of BPF (50 mg/kg), concurrently with surgery (day 0), prevented MnSOD acetylation. In these conditions, prohibitin or MnSOD expression was statistically the same among the lanes. Gels are representative of results from 6 animals. Densitometric analyses of all animals per groups are reported MnSOD and acetylated MnSOD were first normalized with prohibitin and then these values were used to obtain acetylated/total MnSOD ratio. Results are expressed as mean ± SEM for 6 rats. * *p* < 0.05 versus sham; ^†^ *p* < 0.05 versus vehicle + CCI.

In particular, it is well known that the main targets of SIRT3 is the MnSOD which detoxifies the radicals generated in the mitochondrial respiratory chain [38,58]. In this regard, we observed an increase in MnSOD acetylation levels either 14 or 21 days after CCI (Figure 10), at the same time as SIRT3 inactivation.

**Figure 11:**
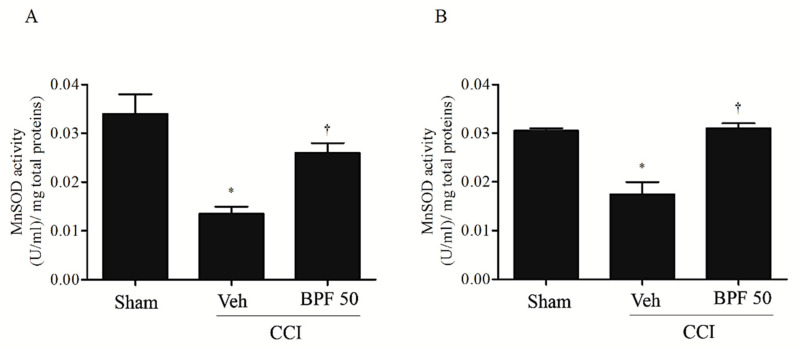
Acetylation on MnSOD is associated to inactivation of its biological function. MnSOD acetylation is intimately linked to inactivation of MnSOD enzymatic function either 14 days (**A**) and 21 days (**B**) post injury. In fact, when compared with sham group, CCI rats showed low levels of MnSOD activity. Subcutaneous infusion of BPF (50 mg/kg), concurrently with surgery (day 0), prevented mitochondrial proteins acetylation. Results are expressed as mean ± SEM for 6 rats. * *p* < 0.05 versus sham; ^†^ *p* < 0.05 versus vehicle + CCI.

This event is associated with the inhibition of MnSOD activity (Figure 11). Treatment with BPF (50 mg/kg), most probably, prevented MnSOD acetylation restoring its activity (Figure 11).

## 4. Discussion

Chronic pain is one of the most serious public health problem. Although the huge amount of studies concerning this pathology, nowadays there is not a precise treatment for chronic pain [59]. Moreover, the lack of specific diagnostic tools makes chronic pain therapy really arduous [60,61] increasing the gap between preclinical and clinical results.

Toxic agents, such as reactive oxygen species (ROS), are involved in the development and maintenance of various diseases, including neuropathic pain [20,23,48,49], regulating activation of N-methyl-d-aspartate (NMDA) receptor [62] and then leading to the development of hyperalgesia [20,48]. 

Considering that mitochondrial dysfunctions are linked to pain, the protection of mitochondrial functions against oxidative stress can be considered as a therapeutic strategy to alleviate or prevent chronic pain states [26,62,63,64,65].

The discovery of sirtuins (SIRTs) involvement in pain pathways and their dysfunctional modulation by free radical products, could be of great interest for the identification of new potential therapeutic targets for the management of the different etiologies of pain. Particularly SIRT3, already involved in the regulation of cellular energy metabolism, in cardiovascular diseases and in the control of apoptosis [42,66], has recently emerged as a novel regulator of mitochondrial metabolism and function too [42,66].

In this study, we showed that the development of hyperalgesia and allodynia after CCI of the sciatic nerve in rats (Figure 1), was associated with the increase of MDA and 4-HNE levels (biochemical markers of lipid peroxidation and oxidative stress) and attenuated by treatment with BPF at 50 mg/kg (Figure 2, Figure 3 and Figure 4). Moreover, during neuropathic pain induced by CCI, we showed an increase of SIRT3 carbonylation (Figure 6) and then its inactivation (Figure 7), responsible for the mitochondrial hyperacetylation event (Figure 8 and Figure 9), blocked by treatment with BPF (50 mg/kg). Our data suggest that lipid peroxidation products play a key role in the generation of neuropathic pain and, taken together, these results show that free radicals and 4-HNE are important mediators of the induction of hyperalgesia and allodynia during chronic pain, via modulation of mitochondrial activities. This action was pharmacologically reverted through the utilization of antioxidants.

Oxidative/carbonyl stress-induced different effects on SIRT3, such as the decrease of its activity, responsible for acetylation of MnSOD (Figure 10). The consequent deactivation of this protective enzyme, enhancing hyperalgesia and allodynia, would lead to a vicious circle that could contribute to the neuropathic pain insurgence and maintenance.

Therefore, inhibition of SIRT3 carbonylation by polyphenols (Figure 6) could be beneficial for therapeutic intervention of chronic pain and hyperalgesia modulation, observed during neuropathic pain [19]. Numerous studies have now observed that polyphenols have antioxidant and anti-inflammatory properties and their ability to activate sirtuins, either directly or indirectly, has also been described in a variety of models [67,68,69,70].

Recently, the interest in natural antioxidants as pharmacological drugs, in several diseases, is increased [31,32] since their utilization could be considered as an alternative affordable approach against the insurgence and the development of pathology.

Epidemiological studies have evidenced beneficial effects in humans [71], such as the prevention of degenerative diseases (e.g., cancer), cardiovascular diseases [72], neurodegenerative diseases, diabetes or osteoporosis [73].

Moreover, it has been observed that administration of BPF, hydroxytyrosol, oleuropein or resveratrol was able to attenuate the allodynia and the hyperalgesia through the removal of ROS/RNS in animal models of pain of different etiologies [19,21,31,32,54].

## 5. Conclusions

Our findings demonstrated that restoring mitochondrial functions and protecting SIRT3 activity by natural antioxidants, could be beneficial during oxidative stress-induced allodynia and hyperalgesia. Besides, re-establishing the activity of SIRT3 by BPF would be a new target in therapeutic intervention for the management and rehabilitation of pain suffering patients.

## Supplementary Materials

The following are available online at https://www.mdpi.com/2076-3921/9/11/1103/s1, Figure S1. Effects of treatment with BPF or pregabalin on allodynia and hyperalgesia after inducing CCI in rats, Figure S2. The development of hyperalgesia and allodynia coincided with an increase of 4-HNE levels either 14- or 21-days post injury, Figure S3. The inhibition of SIRT3 induces acetylation on mitochondrial proteins either 14- or 21-days post injury.
