# Supplementary material for: Natural Antioxidant Control of Neuropathic Pain—Exploring the Role of Mitochondrial SIRT3 Pathway
Source: Antioxidants (Basel). 2020 Nov 9;9(11):1103. doi: 10.3390/antiox9111103 (PMC7698145; doi:10.3390/antiox9111103)
Supplement: Supplementary file 1 [file antioxidants-09-01103-s001.pdf]

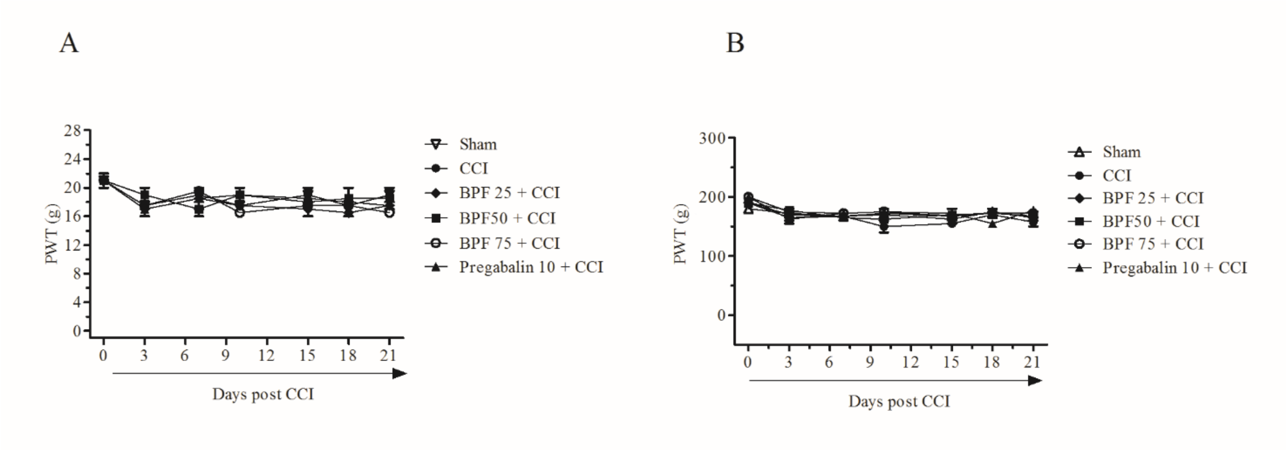

Figure 1. Effects of treatment with BPF or pregabalin on allodynia and hyperalgesia after inducing CCI in rats. No differences were detected in the contralateral paw behaviors in all groups during mechanical allodynia (**A**) or hyperalgesia (**B**). Data are expressed as mean ± SEM for 15 rats in each group. PTW: paw withdrawal threshold.

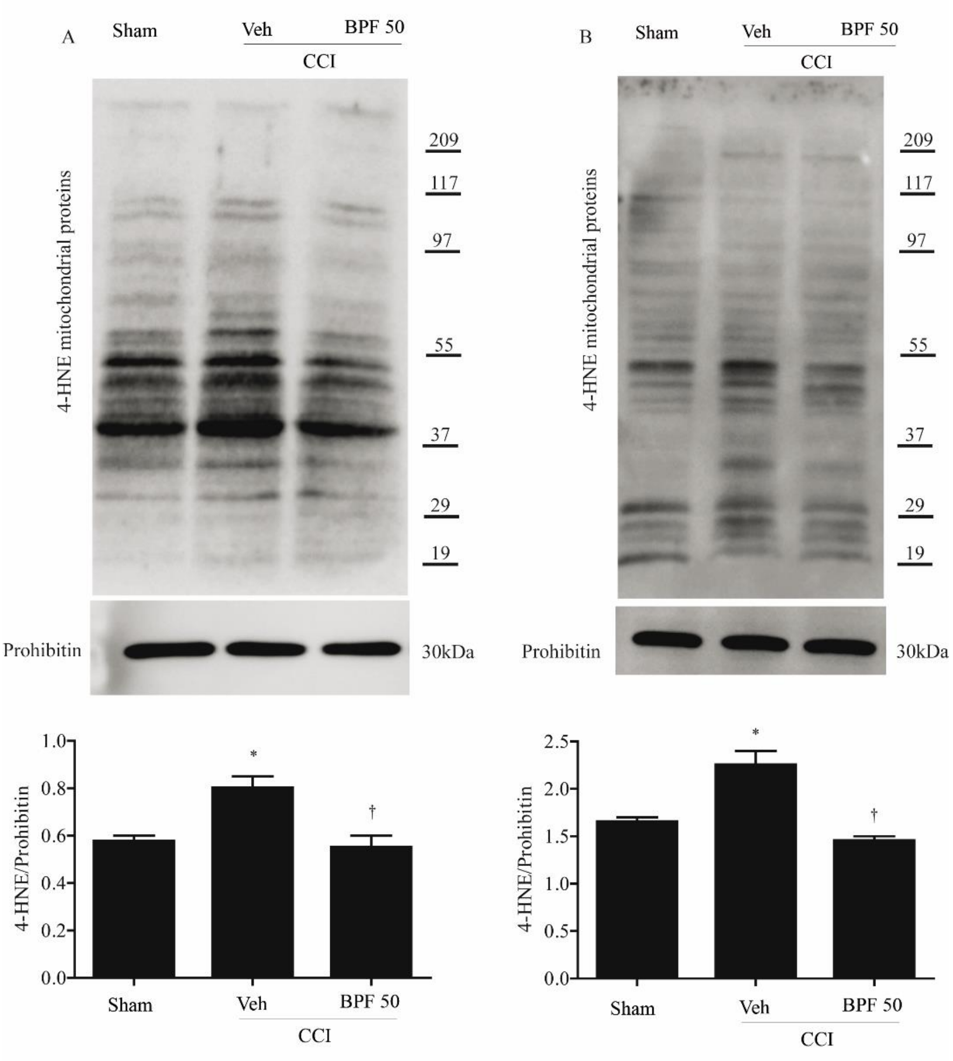

Figure 2. : The development of hyperalgesia and allodynia coincided with an increase of 4-HNE levels either 14 and 21 days post injury. CCI rats showed a significant production of 4-HNE in spinal cord

either 14 days (**A**) and 21 days (**B**) post injury as measured by Western Blot. Treatment with BPF (50 mg/kg) induced a significant reduction of these values. (**A**,**B**) Gels are representative of results from 6 different animals. (**A**,**B**) No difference for prohibitin expression was detected among the lanes in these conditions. Densitometric analyses of all animals per groups are reported. Results are expressed as mean ± SEM for 6 rats. * $p < 0.05$ compared to sham; $^{\dagger}p < 0.05$ compared to veh + CCI.

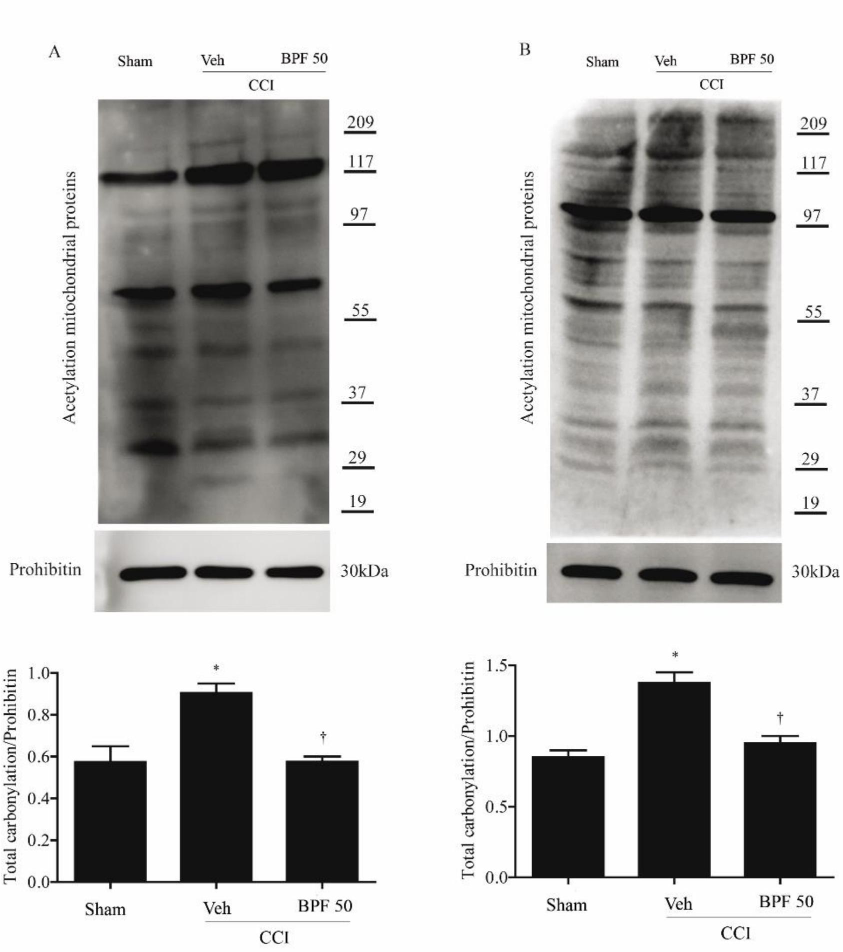

Figure 3: The inhibition of SIRT3 induces acetylation on mitochondrial proteins either 14 and 21 days post injury. Chronic constriction injury of the sciatic nerve in rats led to significant acetylation of mitochondrial proteins either 14 days (**A**) and 21 days (**B**) post injury. Treatment with BPF (50 mg/Kg) prevented mitochondrial proteins acetylation. No difference for prohibitin expression was detected among the lanes in these conditions. Gels are representative of results from 6 animals. Densitometric analyses of all animals per groups are reported. Results are expressed as mean ± SEM for 6 rats. * $p < 0.05$ compared to sham; $^{\dagger}p < 0.05$ compared to veh + CCI.
